# AI-assisted peer review: efficiency for reviewers, burden for authors—a narrative review

**DOI:** 10.3389/frai.2026.1968385

**Published:** 2026-10-06

**Authors:** Asaad Ma Babker

**Affiliations:** Department of Medical Laboratory Sciences, College of Health Sciences, Gulf Medical University, Ajman, United Arab Emirates

**Keywords:** algorithmic bias, artificial intelligence, author burden, editorial accountability, large language models, peer review, research integrity, scholarly publishing

## Abstract

Artificial intelligence (AI) in scholarly peer review has the potential to simplify the work of editors and reviewers through faster triage, automated reporting completeness checks, and scalable synthesis of reviewer comments. However, it also presents practical, ethical, and technical limitations that may place implementation burdens on authors. I examine bias arising from training data and model architecture, confidentiality risks from commercial AI deployment, accountability gaps when human judgment is displaced, and the growing threat of hallucinated citations to scientific integrity. I further analyze how poorly governed AI integration shifts remediation and formatting burdens onto authors—particularly those from under-resourced or non-Western institutions. I propose hybrid human–AI workflows, mandatory disclosure standards, and equity-conscious implementation frameworks to address these harms. Responsible AI integration in peer review requires transparency, human accountability, and deliberate protection of the diversity of voices represented in scientific literature. These considerations also extend beyond individual actors to recursive feedback loops in which AI-assisted manuscripts are evaluated by AI-assisted review systems, reinforcing the need for human contextual stewardship.

## 1 Introduction

The rapid advancement of artificial intelligence (AI) has transformed various aspects of scientific research, including academic publishing and peer review. Large language models and other AI tools have proven in recent years to be capable of automating many tasks that have historically been performed by human editors and reviewers. Automated language and grammar checks, plagiarism detection, format compliance, and even preliminary evaluation of research significance are some of these applications (Doskaliuk et al., 2025).

Early implementations indicate that AI can effectively detect common omissions, highlight internal inconsistencies, and produce structured summaries that assist editors and reviewers, potentially increasing consistency and throughput (Dore et al., 2026). However, these seeming efficiencies come with trade-offs that may make it more difficult for authors to implement their manuscripts. In addition to creating incentives to optimize manuscripts for algorithmic rather than scientific criteria and producing opaque or inconsistent evaluations when models hallucinate, drift across versions, or are applied unevenly across review workflows, tools that streamline reviewer tasks may shift work downstream, imposing additional formatting, reporting, or remediation demands on authors (May et al., 2026).

AI-assisted review raises significant issues regarding bias, confidentiality, accountability, and reproducibility in addition to burdens on authors (Ebadi et al., 2025). Public model use can result in confidentiality violations if manuscripts are exposed to third-party training pipelines; proprietary model opacity and prompt sensitivity hinder auditability and reproducibility; and models inherit biases from training data and architectures that can distort assessments by topic, methodology, or population (Haibe-Kains et al., 2020). Major editorial organizations and publishers have responded to these problems by issuing guidelines that limit the unrestricted use of AI in authorship and peer review and by advocating for human accountability, transparency, and safe deployment models (Wang and Gong, 2026).

However, integrating AI into peer reviews raises concerns regarding transparency and accountability. Critics argue that the opacity of AI systems can lead to new forms of bias, including confidentiality compromises and limitations in handling complex academic work, which might undermine the review process (Leung et al., 2023).

Because automated checks frequently produce time-consuming requests (reformatting data, providing code/notebooks, or rerunning analyses), sometimes based on false positives or unclear rationale, AI can make implementation more difficult for authors by shifting technical and administrative burdens onto them. When AI outputs lack provenance or regular editorial oversight, this leads to an increase in workload, delays, and injustices for researchers with limited resources or support.

Beyond its role in scholarly communication, AI has achieved transformative penetration across clinical medicine, further underscoring the imperative for rigorous evaluation of AI-generated outputs at every stage of scientific knowledge production. In diagnostic medicine, AI-driven tools now operate across hematology laboratories, radiology suites, and pathology workflows, with recent evidence documenting their capacity to detect morphological abnormalities, flag laboratory errors, and assist in risk stratification—while simultaneously raising medicolegal concerns around clinical accountability, failure mode transparency, and the governance of algorithmic decision-making in high-stakes diagnostic environments (Babker, 2026). In pharmacology and therapeutics, AI is reshaping drug discovery and repurposing pipelines: computational models guided by machine learning have demonstrated the capacity to identify candidate molecules, predict off-target effects, and accelerate translational pathways from bench to bedside, exemplified by recent AI-guided drug repurposing frameworks that integrate computational innovation with experimental validation to address unmet oncological needs (Fawzy et al., 2026). AI is also being applied to the management of complex infectious diseases, including HIV, where personalized medicine approaches informed by AI algorithms are being evaluated for their potential to optimize antiretroviral regimens, predict treatment outcomes, and tailor interventions to individual patient profiles (Sah et al., 2025). The rapid clinical embedding of AI across these domains means that the manuscripts now entering peer review are themselves increasingly AI-assisted in their conduct—creating a compounding challenge in which AI tools evaluate research that was partly produced by AI, with implications for the detection of methodological artifacts, the verification of computational claims, and the integrity of the scientific record. This coupled use of AI in manuscript production and evaluation can create a recursive system-level feedback loop, making human contextual stewardship—ground-truth verification, ethical judgment, communication of uncertainty, and recognition of meaningful anomalies—particularly important (Biele and Kowalski, 2026).

This critical narrative review synthesizes empirical studies, conference deployment reports, editorial guidelines, publisher policies, and expert commentaries published between January 2020 and March 2026 to address four interconnected objectives: first, to evaluate how accurately AI tools detect methodological and reporting issues relative to human reviewers; second, to characterize the ethical, confidentiality, and reproducibility risks arising from AI integration in peer review workflows; third, to assess current validation strategies and their adequacy for ensuring AI accountability and performance transparency; and fourth, to identify workable implementation recommendations that preserve editorial responsibility and equity in scholarly publishing—with particular attention to the unintentional but measurable shifting of remediation and administrative burden onto authors, especially those from under-resourced or non-Western institutions.

## 2 Methods: literature search and selection strategy

This critical narrative review synthesizes current evidence, guidelines, and perspectives on AI integration in scholarly peer review, with emphasis on performance evaluation, ethical implications, validation approaches, and impacts on author workload. A narrative rather than systematic approach was selected given the heterogeneity of source types, the rapid evolution of the field, and the breadth of thematic domains addressed—conditions under which thematic synthesis offers greater analytical flexibility than formal systematic methods.

Literature was identified through searches of PubMed/MEDLINE, Web of Science, Scopus, and Google Scholar, covering January 2020 through March 2026. Search terms combined concepts related to artificial intelligence (including large language models and natural language processing), peer review and editorial processes, and outcomes including performance, bias, ethics, and author burden. Supplementary searches targeted specific AI tools applied in peer review contexts (including ChatGPT, iThenticate, and statcheck) and policy documents from major publishers and editorial organizations including the Committee on Publication Ethics (COPE), the International Committee of Medical Journal Editors (ICMJE), and the World Association of Medical Editors (WAME). Preprint servers (arXiv and bioRxiv) were additionally searched to capture emerging evidence given the recency of developments in this field.

Sources were selected based on direct relevance to AI use within peer review workflows. Included material encompassed empirical studies, randomized and observational evaluations, conference deployment reports, expert commentaries, implementation case reports, and official organizational guidelines. Material focused solely on AI in research conduct or on AI-generated content unconnected to review processes was excluded, as were non-English publications without available translations.

Given the narrative design, sources were appraised for relevance, methodological contribution, and thematic fit rather than through formal quality-scoring instruments. The primary search focused on January 2020 through March 2026; earlier seminal methodological studies identified through citation chaining and reviewer-suggested references were retained when directly relevant to computational peer review. Following deduplication and revision, 51 unique sources were retained. Conflicting empirical findings were not pooled or resolved by majority vote. Greater interpretive weight was given to peer-reviewed empirical studies that evaluated AI against human reviewers or predefined reference standards and reported sufficiently transparent methods. Where findings diverged, differences were retained and interpreted in relation to the model or version tested, the review task, prompting strategy, dataset, and study design. Policy documents and expert commentaries were used to inform governance and implementation issues rather than as substitutes for empirical performance evidence. The revised synthesis prioritizes peer-reviewed published evidence; preprints were replaced where a suitable published source was available. Findings were synthesized thematically across five key domains: AI performance metrics, ethical risks, validation strategies, reviewer-facing benefits, and author-facing burdens.

## 3 Peer review workflow and AI intervention points

Rather than restating the conventional peer-review sequence, the relevant issue for this review is where AI can enter the workflow and how its use changes responsibility and risk (Figure 1) (Santini, 2018; Tumin and Tobias, 2019; Walshe and Ashby, 2026). At submission and editorial screening, computational systems can support text-similarity checking, reporting and formatting checks, manuscript classification, and structured triage. Earlier AI literature also describes computational support for reviewer profiling and matching, while workflow simulations show that algorithmic reviewer assignment can shorten review duration under specified assumptions (Kousha and Thelwall, 2024; Shah, 2022; Price and Flach, 2017; Mrowinski et al., 2017).

**Figure 1:**
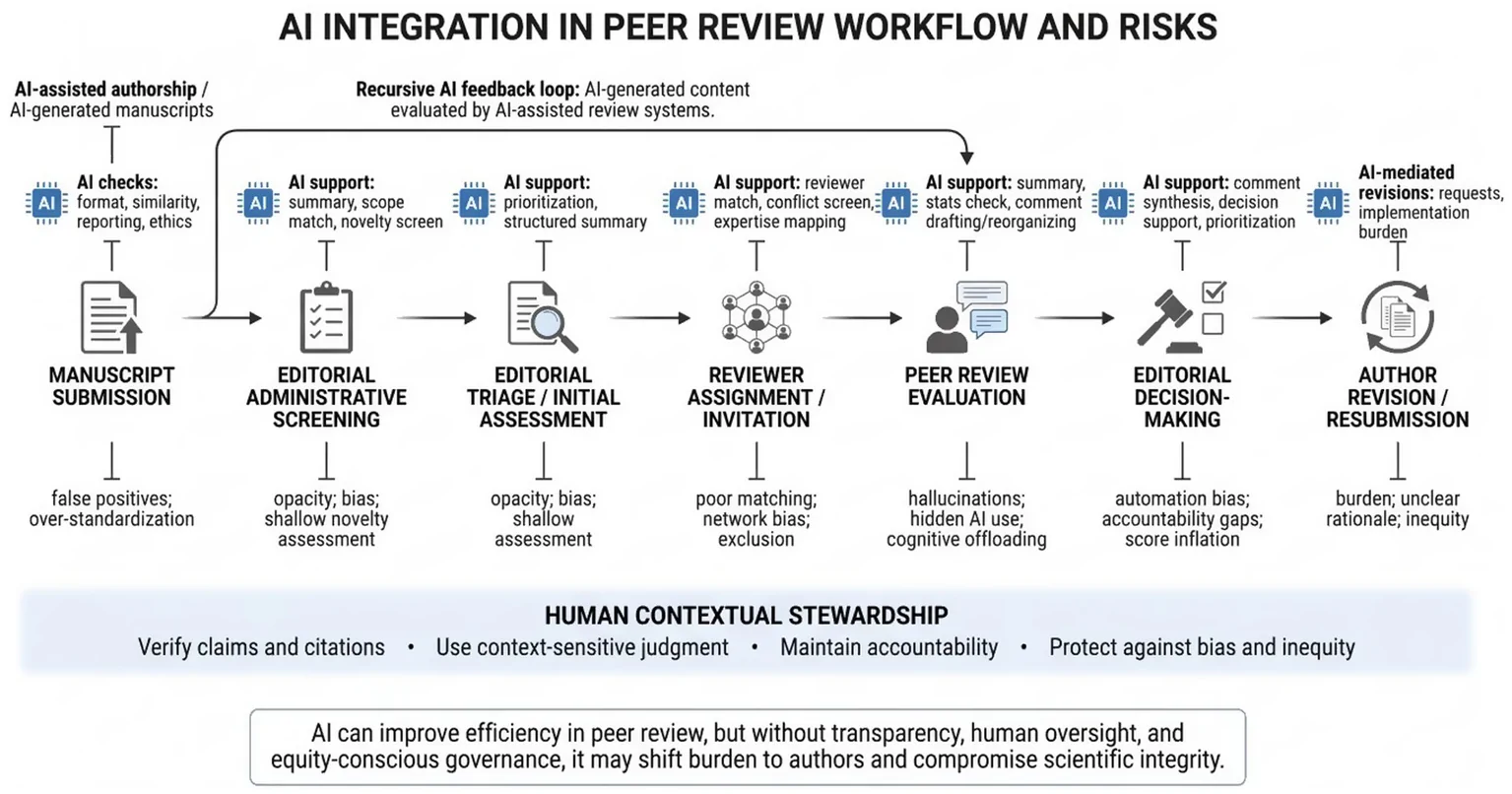
AI integration in the peer-review workflow: intervention points, risks, and safeguards. The figure illustrates seven major stages of the peer-review pathway, from manuscript submission to author revision and resubmission, highlighting where artificial intelligence may support editorial and reviewer activities and the corresponding risks or failure modes at each stage. The recursive feedback loop represents the growing interaction between AI-assisted authorship and AI-assisted peer review, whereby AI-generated or AI-modified manuscripts may themselves be evaluated using AI-supported systems. Human contextual stewardship remains essential across the workflow to verify claims and citations, apply context-sensitive scientific judgment, maintain accountability, and protect against bias and inequity. This figure was created by Biorender.

During substantive review, large language models can summarize manuscripts, organize or draft comments, and assess structured reporting criteria. Published empirical studies show, however, that performance is strongly task-dependent: checklist-like tasks may be useful in selected settings, whereas agreement with human reviewers is substantially weaker for methodology and context-dependent judgment (Chia et al., 2026; Suleiman et al., 2024; Saad et al., 2024; Liang et al., 2024b). Computational resources such as benchmark review corpora can support more reproducible evaluation of these systems (Ghosal et al., 2022).

At editorial synthesis and author revision, AI can assist with consolidation of reviewer comments, decision support, and generation of revision prompts. These stages also concentrate important failure modes, including false positives, opaque ranking, hallucinated claims or citations, confidentiality risks, automation bias, cognitive offloading, and transfer of verification or remediation work to authors. AI outputs should therefore function as decision support rather than autonomous adjudication, with final responsibility retained by human editors and reviewers (Leung et al., 2023; Hosseini and Horbach, 2023; National Institutes of Health, 2023; Lauer et al., 2023; Li et al., 2024; Naddaf, 2025; Zou, 2024).

## 4 Evaluation of AI performance in peer review

Evaluating AI performance in peer review demands comprehensive assessment using both quantitative metrics and qualitative judgment (Thomas et al., 2021). A robust evaluation framework must address measurement accuracy, substantive review quality, prospective workflow design, and ongoing iterative monitoring—each examined in turn below.

### 4.1 Quantitative metrics

Quantitative measures encompass accuracy in identifying reporting checklist items, sensitivity and specificity for detecting data inconsistencies or statistical errors, concordance with human reviewer recommendations (accept/revise/reject), and time-to-completion improvements (Sizo et al., 2025). Critical error metrics include rates of hallucinations or fabricated citations, false positives (erroneously flagged problems) and false negatives (missed issues), and reproducibility of AI-generated findings across different model versions and prompt conditions (Adel and Alani, 2025). Evaluation frameworks should monitor downstream effects including editor workload—particularly time spent verifying AI output—and decision turnaround times (Thomas et al., 2021). Published empirical work illustrates why performance should be reported at the task level rather than collapsed into a single “AI accuracy” value. Chia et al. evaluated a generative model against COREQ criteria across 15 qualitative studies and found markedly heterogeneous criterion-level performance, with 15 of 32 criteria classified as information-limited (Chia et al., 2026). In a field experiment using conference abstracts, Shcherbiak et al. likewise reported low agreement between human and AI quality assessments overall, despite closer alignment for the highest-quality abstracts (Shcherbiak et al., 2024). These findings caution against generalizing performance from one review task to another.

### 4.2 Qualitative assessment

Qualitative assessment must examine the substantive value of AI contributions, including relevance and insight of critiques, ability to evaluate novelty and clinical significance, and sensitivity to contextual factors such as study settings or equity considerations. Human reviewers and editors should systematically rate AI comments for usefulness, specificity, and potential to enhance manuscript quality (Lemberger et al., 2026). Attention should focus on bias audits examining topic-, methodology-, geographic-, gender-, and race-related biases, and whether AI feedback systematically favors certain research paradigms while marginalizing context-specific insights from under-represented methodological traditions (Hosseini and Horbach, 2023). Direct comparison with human review also shows this limitation. Suleiman et al. assessed 720 human-review comments across 20 medical research articles and found complete agreement with GPT-4 for 6.7% of comments, partial agreement for 12.8%, no agreement for 78.5%, and contradiction for 2.1%, with particularly low agreement for methods (Suleiman et al., 2024). Fairness is also prompt-sensitive: in a randomized trial of AI-assisted reviewer identification, GPT-4o recommendations without an explicit diversity, equity, and inclusion prompt were predominantly male (68%) and from high-income countries (95.3%); explicit prompting substantially changed both gender and geographical representation (Teixeira, 2025).

### 4.3 Prospective evaluation frameworks

Evaluation should occur within realistic, prospective workflows rather than relying solely on curated retrospective datasets. Parallel trials comparing editor decisions informed by human review alone, AI review alone, and hybrid AI–human workflows will provide the most robust evidence of benefits or potential harms. Outcome measures should encompass both process metrics—speed and completeness of evaluations—and scientific-quality metrics, including manuscript quality improvements and incidence of post-publication corrections or retractions. Safety monitoring must include comprehensive confidentiality risk assessments ensuring that models or deployment mechanisms do not compromise unpublished data security (McAlister et al., 2025). Prospective or parallel designs should also examine whether AI assistance changes author revisions and editorial decisions, rather than measuring textual similarity alone. In an observational study of 21 published articles, Saad et al. found meaningful overlap between ChatGPT and human reviews but concluded that fully automated peer review could not be recommended (Saad et al., 2024). Comparative studies in surgical manuscripts likewise demonstrate differences between AI and human review behavior, supporting hybrid rather than replacement models (Marrella et al., 2025; Joachim et al., 2025).

### 4.4 Iterative monitoring

Evaluation must remain iterative and transparent given the rapid evolution of AI models and prompts. This requires continuous performance monitoring, versioned reporting of evaluation results, and clear disclosure of AI use to authors and reviewers (Chia et al., 2026). When deficiencies are identified—including confabulation, biased recommendations, or cognitive offloading, whereby reviewers place excessive and uncritical trust in AI-generated outputs—journals should refine model access, restrict high-risk tasks, and maintain human accountability for final judgments. Only through empirical, ongoing study within accountable human-in-the-loop systems can AI tools be responsibly integrated into peer review processes. Cross-platform evidence reinforces the need for repeated validation: Thelwall and Yaghi found that ChatGPT’s ability to predict peer-review outcomes varied substantially across F1000Research, SciPost Physics, and ICLR datasets and with prompt/input conditions, indicating that performance estimates cannot be assumed to transfer across venues or workflows (Thelwall and Yaghi, 2025).

## 5 Ethical risks of AI in peer review

The use of AI in peer review raises several ethical risks that journals must address. Chief among these is confidentiality: uploading unpublished manuscripts to external models can expose sensitive data, patient information, and intellectual property, and may feed training datasets without consent.

### 5.1 Confidentiality and data security

A foundational concern is the breach of manuscript confidentiality. In June 2023, the National Institutes of Health (NIH) released Guide Notice NOT-OD-23-149, prohibiting the use of generative AI technologies in the NIH peer review process. The policy’s intent is to maintain security and confidentiality, since using generative AI requires sharing application materials, which violates NIH’s existing confidentiality requirements. NIH accordingly revised its Security, Confidentiality, and Non-disclosure Agreements for Peer Reviewers to explicitly prohibit using AI tools to analyze or critique grant applications and R&D contract proposals. The underlying rationale is straightforward: once information is loaded onto a generative AI platform, there is no guarantee of where that data is being sent, saved, viewed, or used in the future (National Institutes of Health, 2023; Lauer et al., 2023). Uploading unpublished manuscripts to commercial AI systems therefore risks exposing sensitive findings and proprietary data without authors’ knowledge or consent.

### 5.2 Accountability and responsibility

The adoption of AI raises key ethical issues around accountability, responsibility, and transparency. The Committee on Publication Ethics (COPE) has consistently maintained that AI tools cannot fulfil authorship or decision-making roles, as authorship requires accountability, informed consent, and intellectual contribution—qualities AI tools do not possess. This principle applies equally to peer review: when evaluative judgment is delegated to AI, the lines of human responsibility for editorial decisions become obscured, undermining the ethical foundations of the review process (COPE Council, 2024).

### 5.3 Bias and inequity

Hosseini and Horbach (2023) examined how large language models (LLMs) such as ChatGPT could affect peer review, raising concerns about bias, research integrity, and equality of access (Hosseini and Horbach, 2023). AI models trained on vast human-generated text—which frequently includes author identities and institutional affiliations from publicly available papers—may perpetuate or even amplify existing biases present in that training data. Whether AI-based reviewers reduce bias by focusing solely on textual content or exacerbate it by incorporating learned patterns about authors and institutions remains an open and largely unexplored question. Models trained predominantly on English-language, Western-institution data risk systematically disadvantaging research from under-represented regions or methodological traditions that are sparse in training corpora (Hosseini and Horbach, 2023).

### 5.4 Fabrication and confabulation

Fabrication and confabulation remain material risks when LLM-generated content is treated as evidence without independent verification. In a published evaluation of 636 citations generated across 84 ChatGPT-produced literature reviews, Walters and Wilder found that 55% of GPT-3.5 citations and 18% of GPT-4 citations were fabricated; among citations to real works, substantive citation errors were also common (43 and 24%, respectively) (Walters and Wilder, 2023). Although this study evaluated generated literature reviews rather than peer-review reports, it provides direct empirical evidence for the citation-verification problem that becomes relevant when reviewers or editors use LLMs to generate literature-based critiques. Peer-review-specific published estimates of hallucinated-citation frequency remain limited, so the magnitude of this risk within journal review workflows should not be overstated.

### 5.5 Undisclosed use and fairness

The use of LLMs in an undisclosed manner in peer review poses a significant ethical concern because authors and editors may be unable to distinguish human judgment from model-generated text. A large-scale analysis of ICLR 2024, NeurIPS 2023, CoRL 2023, and EMNLP 2023 reviews estimated that between 6.5 and 16.9% of submitted review text could have been substantially modified by LLMs beyond spell-checking or minor language editing (Liang et al., 2024a). The estimated fraction was higher in reviews reporting lower confidence, submitted close to deadlines, and from reviewers less likely to respond to author rebuttals (Liang et al., 2024a). At the journal level, policy remains heterogeneous: a cross-sectional study of the top 100 medical journals found substantial variation in guidance governing AI use during peer review (Li et al., 2024). Unequal access to advanced AI tools may further compound fairness concerns, reinforcing the need for explicit disclosure, confidentiality safeguards, and human accountability.

### 5.6 Mitigation

To address these risks, journals should implement strict data-protection protocols, require transparent disclosure of AI use while maintaining full human accountability for editorial decisions, conduct regular bias audits of AI tools, independently verify AI-generated assertions, and ensure meaningful human oversight at every stage of the review process.

## 6 Validation strategies for AI in peer review

Validating AI tools in peer review demands rigorous, multi-dimensional approaches that extend well beyond simple accuracy metrics. A comprehensive validation framework must address prospective workflow design, continuous performance monitoring, hallucination detection, and the human behavioral factors that shape how AI-assisted reviews are produced and received—each examined in turn below.

### 6.1 Prospective parallel workflows

Prospective validation studies should employ parallel workflows comparing human-only, AI-assisted, and hybrid AI–human review processes and should measure both efficiency and substantive review quality. Published comparative studies illustrate why. Suleiman et al. found that most human reviewer comments had no equivalent in GPT-4 output, particularly for methodological issues (Suleiman et al., 2024). Saad et al. observed partial convergence between ChatGPT and human reviewers but did not support fully automated review (Saad et al., 2024). Studies in hand surgery and oral and maxillofacial surgery similarly identified meaningful differences between AI-generated and human peer review behavior (Marrella et al., 2025; Joachim et al., 2025). Taken together, the published evidence supports task-specific assistance and human–AI complementarity rather than assuming that agreement on an editorial outcome is sufficient evidence of review quality.

### 6.2 Continuous monitoring and bias audits

Journals must establish continuous monitoring systems that track model performance across versions and assess inter-rater reliability between AI and human reviewers. A large study covering more than 5,000 papers from Nature journals, ICLR, and eLife found that GPT-4 feedback overlapped with that of an individual human reviewer by approximately 30.9% for Nature-journal submissions, compared with 28.6% human-to-human overlap, and by 39.2% for the ICLR dataset versus 35.3% human-to-human overlap (Liang et al., 2024b). However, such aggregate overlap does not establish equivalence in methodological judgment. Bias auditing should also be prompt-sensitive: Teixeira’s randomized trial found that GPT-4o reviewer recommendations were strongly skewed toward male scientists and high-income-country affiliations without a diversity prompt, while explicit diversity prompting substantially changed the distribution of recommended experts (Teixeira, 2025). Model versions, prompts, performance metrics, and known limitations should therefore be documented and periodically re-evaluated as systems evolve (Naddaf, 2025; Zou, 2024).

### 6.3 Hallucination detection

Validation protocols must include systematic hallucination detection to verify AI-generated citations and factual claims before they propagate through the review process. Direct published estimates of hallucinated citations specifically within journal peer-review reports remain scarce. Broader empirical evidence nevertheless demonstrates the underlying risk: Walters and Wilder documented high rates of fabricated and erroneous citations in LLM-generated literature reviews (Walters and Wilder, 2023). Journals using AI in peer review should therefore verify cited works and factual claims independently rather than treating model-produced references as self-validating, while avoiding claims about peer-review-specific hallucination rates until direct evidence is available.

### 6.4 Human factors and cognitive offloading

Validation must also extend to the human behavioral dimensions of AI-assisted review, because efficiency gains at the system level could be offset by degraded judgment at the individual level. Evidence specific to peer reviewers remains limited. In a mixed-methods study of 666 participants, Gerlich reported a negative association between frequent AI-tool use and critical-thinking ability mediated by increased cognitive offloading, but the study was not conducted in a peer-review setting (Gerlich, 2025). Cognitive offloading should therefore be treated as a plausible human-factors risk requiring direct prospective study in reviewers, rather than as an established peer-review effect. Disclosure and meaningful human oversight remain important safeguards (Naddaf, 2025; Zou, 2024).

## 7 How AI simplifies reviewing for reviewers

The integration of artificial intelligence into the peer review workflow offers reviewers a meaningful opportunity to reduce procedural burden while enhancing the overall quality and depth of manuscript evaluation. By automating mechanistically demanding and time-intensive tasks, AI technologies empower reviewers to allocate greater cognitive resources toward higher-order evaluative functions—including appraisal of scientific novelty, clinical or theoretical significance, and nuanced interpretation of methodologically complex results. The principal domains in which AI currently simplifies reviewer work are outlined below.

### 7.1 Reporting compliance screening

AI-powered screening systems can conduct systematic preliminary assessments, verifying manuscript compliance with internationally recognized reporting guidelines—including the Consolidated Standards of Reporting Trials (CONSORT), the Preferred Reporting Items for Systematic Reviews and Meta-Analyses (PRISMA), and the Strengthening the Reporting of Observational Studies in Epidemiology (STROBE)—alongside confirmation of mandatory components such as ethics declarations, data availability statements, and appropriate statistical reporting frameworks (Kousha and Thelwall, 2024; Zhuang et al., 2025). By handling these administrative compliance checks automatically, such tools enable reviewers to redirect their expertise toward substantive scientific appraisal rather than procedural verification, meaningfully reducing the time burden associated with initial manuscript screening.

### 7.2 Structured summarization

Large language models demonstrate considerable capacity for generating structured analytical summaries of complex manuscripts, distilling key findings, characterizing methodological frameworks, and prospectively flagging areas warranting critical scrutiny (Liang et al., 2024b; Checco et al., 2021). This substantially reduces the cognitive load associated with initial manuscript orientation and comprehension, allowing reviewers to engage more efficiently with the substantive content of a submission from the outset rather than investing significant time in preliminary reading and organization.

### 7.3 Statistical verification

Automated statistical verification platforms offer systematic detection of numerical inconsistencies, implausible values, erroneous *p*-value calculations, and discordance between reported statistical outputs and authors’ interpretive conclusions—capturing latent errors that may elude detection during conventional manual review (Kousha and Thelwall, 2024; Nuijten et al., 2016). These tools provide reviewers with a reliable first-pass integrity check on quantitative reporting, reducing the risk that statistical errors pass undetected through the review process and strengthening the overall accuracy of editorial evaluation.

### 7.4 Plagiarism and text similarity detection

AI-assisted text similarity and plagiarism detection algorithms provide an additional layer of integrity assurance, enabling comprehensive identification of overlapping content, self-plagiarism, and inappropriate text recycling with a sensitivity and efficiency that surpasses traditional manual screening (Kousha and Thelwall, 2024; Foltýnek et al., 2019). By automating this aspect of integrity review, these tools reinforce journal standards while concurrently alleviating a time-consuming component of reviewer workload that adds limited scientific value to the evaluation process.

### 7.5 Literature contextualization and feedback organization

AI tools facilitate meaningful literature contextualization by supporting automated retrieval of pertinent prior publications, identification of contradictory evidence within the field, and recommendation of potentially overlooked citations, collectively enhancing the analytical depth and scholarly comprehensiveness of reviewer feedback (Zhuang et al., 2025). Natural language processing applications further augment reviewer performance by supporting the systematic organization of evaluative commentary, proposing constructive and professionally appropriate phrasing for critical observations, and ensuring comprehensive coverage of standardized review domains encompassing methodology, statistical analysis, result interpretation, and ethical considerations (Zhuang et al., 2025).

### 7.6 Limitations and caveats

Nevertheless, these efficiency gains must be carefully balanced against substantive risks. Cognitive offloading—the tendency to delegate analytical effort to AI systems—can erode the depth and independence of human judgment over time, with reviewers placing excessive trust in algorithmically generated assessments even when errors are present (Gerlich, 2025). Additionally, the systemic biases inherent to AI models trained predominantly on English-language and Western-institution data risk propagating methodological and geographic prejudices into reviewer feedback (Hosseini and Horbach, 2023). These considerations collectively underscore the irreplaceable role of human judgment and ethical accountability as foundational imperatives within any AI-augmented peer review paradigm (Walshe et al., 2025).

## 8 How AI in peer review complicates implementation for authors

While AI-assisted peer review promises efficiency gains for editors and reviewers, it can simultaneously create significant implementation burdens for authors—particularly those from under-resourced or non-Western institutions. Beyond administrative and formatting demands, authors face a deeper and more consequential set of complications rooted in confidentiality risks, algorithmic bias, intellectual property vulnerability, and systemic epistemic inequity, each of which is examined below.

### 8.1 Confidentiality and intellectual property risks

A fundamental concern pertains to manuscript confidentiality. When peer reviewers, editors, or automated submission systems upload unpublished manuscripts—containing original data, novel hypotheses, and proprietary methodological innovations—into commercial large language model platforms or third-party AI screening tools, authors’ intellectual property rights and data privacy protections may be materially compromised, with submitted content potentially incorporated into model training datasets without authors’ knowledge or explicit consent (Leung et al., 2023; National Institutes of Health, 2023; Lauer et al., 2023; Li et al., 2024). This risk is not merely theoretical. Major publishers including Elsevier, Springer Nature, and Wiley have explicitly acknowledged in their AI governance policies that uploading manuscripts to external AI tools may constitute a breach of authors’ confidentiality and proprietary rights—particularly where manuscripts contain personally identifiable information, sensitive participant data, or commercially sensitive findings—yet enforcement mechanisms and author notification protocols remain inconsistently implemented across the publishing landscape (Leung et al., 2023; Li et al., 2024). Compounding this vulnerability, the absence of comprehensive and harmonized governance frameworks means that authors often lack transparent disclosure of which AI tools have been applied to their submissions, how those tools processed their content, whether their manuscripts were retained in external systems, and what data protection measures were in place at each stage of the review pipeline (Leung et al., 2023; Hosseini and Horbach, 2023; Li et al., 2024).

### 8.2 Algorithmic bias and epistemic inequity

Algorithmic bias constitutes an equally serious dimension of author-facing complications, with profound implications for the diversity and integrity of the scientific record. AI models deployed in peer review are predominantly trained on corpora of published literature that disproportionately represent high-income, English-language, Western biomedical, and quantitative research paradigms, embedding within them implicit hierarchies of methodological legitimacy that systematically disadvantage qualitative, mixed-methods, community-based participatory, and context-specific approaches from under-resourced or non-Western academic traditions (Hosseini and Horbach, 2023; Teixeira, 2025; Sabet et al., 2023). When such tools assess methodological rigor, evaluate novelty claims, or generate structured revision requests, they may operationalize these embedded biases as ostensibly objective criteria—flagging ethnographic methods as lacking generalizability, penalizing narrative or interpretive frameworks as insufficiently rigorous, or misclassifying innovative study designs from low- and middle-income country (LMIC) contexts as methodologically deficient—not because of genuine scientific weakness but because training data inadequately represents epistemic diversity (Hosseini and Horbach, 2023; Teixeira, 2025; Sabet et al., 2023). Authors from non-English-speaking countries face compounding disadvantages: survey-based evidence demonstrates that non-native English-speaking researchers already expend substantially greater time and cognitive effort in scientific writing, and when AI systems additionally apply language quality filters calibrated to polished, AI-augmented prose, these authors may face disproportionate rejection signals or revision demands that reflect linguistic rather than scientific deficiencies (Sabet et al., 2023; Amano et al., 2023; Walshe et al., 2024).

### 8.3 Opacity of AI-generated feedback

The opacity of AI-generated feedback further entrenches author disadvantage by creating an asymmetric and non-dialogic communication dynamic fundamentally unlike traditional peer review. Authors receiving AI-mediated revision requests frequently cannot identify the specific model, prompt, version, or evaluative criteria responsible for a given assessment; they lack any mechanism to query the reasoning underlying demands; and they have no recourse to the kind of clarifying exchange that characterizes productive human reviewer interaction (Leung et al., 2023; Hosseini and Horbach, 2023; Li et al., 2024). This opacity is particularly damaging when algorithmic assessments contain errors, misinterpretations, or false positives—flagging correctly reported statistical values as inconsistent, misidentifying methodological transparency disclosures as deficiencies, or generating revision demands based on misclassified content—since authors must invest substantial time documenting and refuting spurious concerns without access to the criteria or thresholds that generated them (Chia et al., 2026; Suleiman et al., 2024). The absence of author feedback mechanisms, burden assessment protocols, and false-positive monitoring within most current AI-assisted peer review implementations means that the cumulative error costs of algorithmic misidentification are borne almost entirely by authors, particularly those from institutions without dedicated research support infrastructure, methodological consultants, or statistical review services.

### 8.4 Toward equity-conscious implementation

Without deliberate equity-conscious design, AI-assisted peer review risks not only complicating implementation for individual authors but entrenching systemic inequities that progressively narrow the diversity of scientific voices, methods, and knowledge systems represented in the global scholarly literature. Responsible implementation requires four foundational commitments: author transparency rights that disclose which AI tools were applied and how; bias auditing of AI tools against diverse research methodologies and geographic contexts; explicit governance of data privacy throughout manuscript processing; and institutional accountability frameworks that assign clear human responsibility for AI-generated editorial decisions (Leung et al., 2023; Hosseini and Horbach, 2023; National Institutes of Health, 2023; Lauer et al., 2023; Li et al., 2024; Teixeira, 2025). Journals that adopt these safeguards will be better positioned to realize the efficiency benefits of AI-assisted review without transferring its costs disproportionately onto the authors least equipped to absorb them.

### 8.5 Systemic feedback loops and contextual stewardship

Author- and reviewer-facing effects should not be treated as separate. Biele and Kowalski describe a system-level feedback loop in which growth of AI-generated or AI-assisted submissions on the “supply side” is met by increasing AI-assisted evaluation on the “demand side” (Biele and Kowalski, 2026). If both production and evaluation are optimized around similar model priors, the system may amplify stylistic convergence, overlook context-specific anomalies, and accelerate errors or epistemic narrowing rather than merely redistribute workload. This framework shifts the governance question from whether AI is efficient at an isolated task to whether the coupled author–reviewer–editor system remains capable of correcting itself. Human reviewers therefore retain a distinct role in contextual stewardship: verifying ground truth, arbitrating ethical questions, communicating uncertainty, and recognizing potentially important anomalies that automated systems may classify as noise (Biele and Kowalski, 2026). These functions should be explicit checkpoints in any AI-assisted workflow (Figure 1).

## 9 Conclusion

AI in peer review offers real efficiency gains for editors and reviewers, but these benefits can impose downstream costs on authors through false positives, algorithmic bias, opaque feedback, and undisclosed AI use. Published evidence of fabricated citations, prompt-sensitive bias, and variable agreement with human reviewers demonstrates that efficiency without accountability can threaten scientific integrity (Walters and Wilder, 2023; Suleiman et al., 2024; Liang et al., 2024a; Teixeira, 2025).

Responsible integration demands transparency about AI use, human accountability for all editorial decisions, and equity-conscious design that prevents burden transferring onto authors least equipped to absorb it. Journals should mandate AI disclosure, conduct regular bias audits, and establish author feedback mechanisms to monitor false-positive burdens.

Without these safeguards, AI-assisted peer review risks narrowing the diversity of voices, methods, and knowledge systems that science depends upon. AI can strengthen peer review—but only when governed with the same rigor science demands of the research it evaluates. Governance must also address the recursive author–AI–reviewer feedback loop at the system level, preserving human contextual stewardship as a deliberate safeguard against self-reinforcing errors and epistemic narrowing (Biele and Kowalski, 2026).
